# Prevalence of Chronic Kidney Disease Among Black Individuals in the US After Removal of the Black Race Coefficient From a Glomerular Filtration Rate Estimating Equation

**DOI:** 10.1001/jamanetworkopen.2020.35636

**Published:** 2021-01-29

**Authors:** Jennifer Bragg-Gresham, Xiaosong Zhang, Dao Le, Michael Heung, Vahakn Shahinian, Hal Morgenstern, Rajiv Saran

**Affiliations:** 1 Division of Nephrology, Department of Internal Medicine, University of Michigan, Ann Arbor; 2 Kidney Epidemiology and Cost Center, University of Michigan, Ann Arbor; 3 Morsani College of Medicine, University of South Florida, Tampa; 4 Department of Urology, University of Michigan, Ann Arbor; 5 Department of Epidemiology, University of Michigan, Ann Arbor; 6 Department of Environmental Health Sciences, University of Michigan, Ann Arbor

## Abstract

This cross-sectional study examines whether removal of the Black race coefficient from a glomerular filtration rate (GFR) estimating equation is associated with a change in the estimated prevalence of chronic kidney disease (CKD) in the general Black population and among Black veterans in the US.

## Introduction

The use of a correction for Black race in glomerular filtration rate (GFR) estimating equations for Black adults has recently been challenged on the basis of race being a social construct,^1^ with potential for race-based equations to perpetuate disparities between Black individuals and non-Black individuals.^2,3,4^ Current GFR estimating equations were developed and validated in cohort studies^4^ that included voluntarily participating, representative populations of Black individuals in the US. The coefficient for Black race is an attempt to correct for non-GFR factors associated with serum creatinine concentration. Deleting the coefficient for Black race is associated with an approximately 14% lower estimated GFR (eGFR) among Black patients.^5^ Removal of the coefficient would increase the number of Black individuals being classified as having CKD or reclassified as having a more advanced stage of the disease if they already had the condition. The aim of our study was to assess how much this change at the patient level would affect the distribution of eGFR categories below eGFR of 60 mL/min/1.73 m^2^ (ie, CKD stage 3 or higher, not including dialysis or transplantation) in both the US general population and the population of US veterans who use the Veterans Affairs (VA) Health System.

## Methods

In this cross-sectional study, we analyzed data on 9682 Black adults from nationally representative samples of the US general population from the National Health and Nutrition Examination Surveys (NHANES) from 1999 to 2018 using sampling weights and data on 786 718 Black veterans from the national VA Health System from 2018. Data included were from individuals aged 20 years or older with complete information on race and serum creatinine concentration. No guidelines were used for reporting the data. This research was deemed not regulated without requirement for patient consent by the VA and University of Michigan institutional review boards because it involved public health surveillance through secondary analysis of deidentified data. Research using VA data for this project was approved by the institutional review boards of the University of Michigan and the Ann Arbor VA.

Sample weights used in analyses of NHANES data allowed application of the estimates to the US general population. We estimated a prevalence of an eGFR less than 60 mL/min/1.73 m^2^ among individuals who self-identified as Black in both data sets using the Chronic Kidney Disease Epidemiology Collaboration CKD-EPI equation^5^ with and without the coefficient for Black race. We used SAS, version 9.4 (SAS Institute) for analysis of NHANES data and R, version 3.62 (R Project for Statistical Computing) for VA data.

## Results

The mean eGFR decreased from 102.8 mL/min/1.73 m^2^ (95% CI, 102.1-103.6 mL/min/1.73 m^2^) using the CKD-EPI equation with the race coefficient to 88.1 mL/min/1.73 m^2^ (95% CI, 88.1-89.4 mL/min/1.73 m^2^) using the CKD-EPI equation without the race coefficient in the US adult Black population (mean [SEM] age, 44 [0.24] years; 4260 [44%] male) in NHANES and from a mean (SD) of 82.9 (24.0) mL/min/1.73 m^2^ with the race coefficient to a mean (SD) of 71.6 (21.0) mL/min/1.73 m^2^ without the coefficient among Black US veterans (mean [SD] age, 58.1 [14.3] years; 836 087 [84%] male) (Figure). Elimination of the coefficient for Black race would result in 981 038 (overall prevalence change of 5.8% to 10.4%) more Black individuals being classified as having CKD (eGFR <60 mL/min/1.73 m^2^; ie, CKD stage 3 or higher) in the US adult population (Table). An additional 84 988 (overall prevalence change of 15.5% to 26.3%) Black adults would potentially be classified as having CKD among those using the VA Health System (Table).

**Figure.**
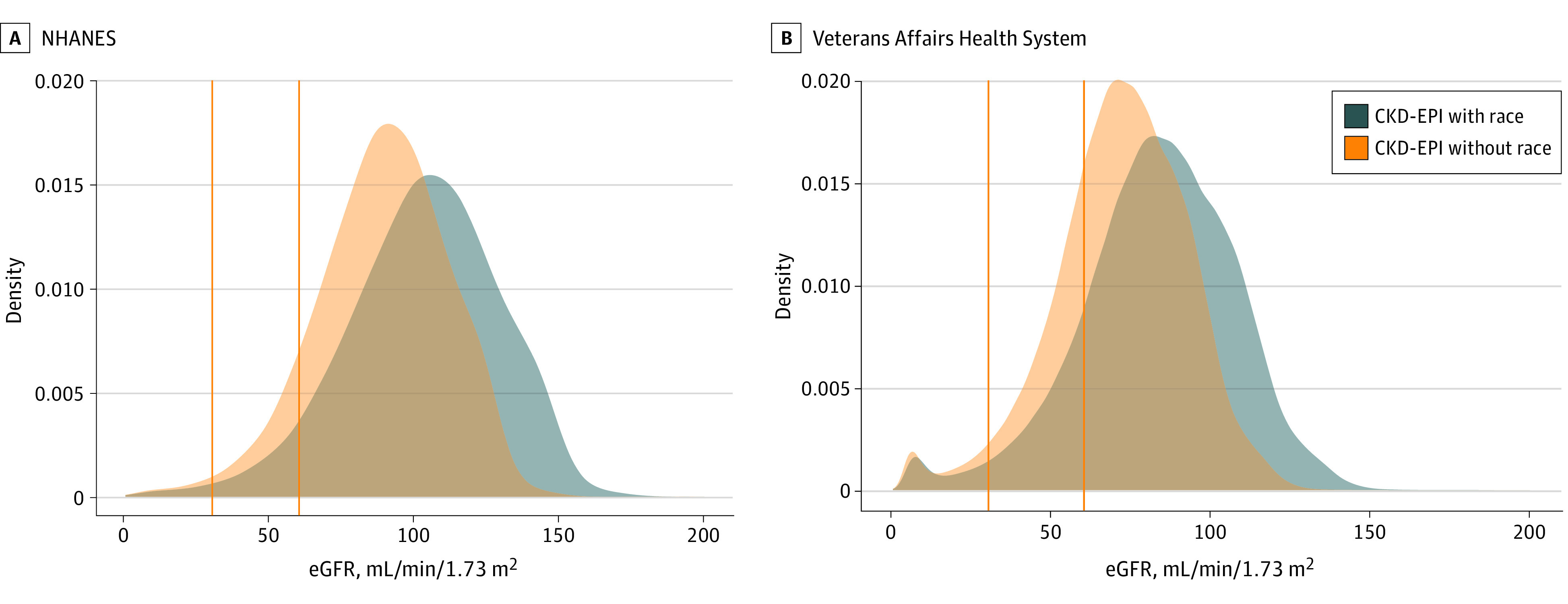
Distribution of Estimated Glomerular Filtration Rate (eGFR) Using the CKD-EPI Equation With and Without the Race Coefficient Among US Black Adults 20 Years or Older A, Data shown are from nationally representative samples of the US general population from the National Health and Nutrition Examination Surveys (NHANES), 1999 to 2018 (n = 9682). B, Data are from the national Veterans Affairs Health System (n = 786 718 Black veterans). The vertical rules represent eGFR cutoffs of 30 and 60 mL/min/1.73 m^3^.

**Table.** eGFR Category Using the CKD-EPI Equation With and Without the Race Coefficient Among Black Adults 20 Years or Older in the US General Population and the VA Health System

eGFR category using CKD-EPI with race coefficient	eGFR category using CKD-EPI with no race coefficient							
	Individuals from NHANES (1999-2018), No. (%)^a^				Veterans from the VA Health System (2018), No. (%)			
	≥60 mL/min/173 m^2^	30-59 mL/min/173 m^2^	<30 mL/min/173 m^2^	Total	≥60 mL/min/173 m^2^	30-59 mL/min/173 m^2^	<30 mL/min/173 m^2^	Total
≥60 mL/min/173 m^2^	19 280 598 (89.6)	981 038 (4.6)^b^	0^b^	20 261 636 (94.2)	579 938 (73.7)	84 988 (10.8)^b^	0^b^	664 926 (84.5)
30-59 mL/min/173 m^2^	0	972 737 (4.5)	67 957 (0.3)^b^	1 040 693 (4.8)	0	93 071 (11.8)	6253 (0.8)^b^	99 324 (12.6)
<30 mL/min/173 m^2^	0	0	208 832 (1.0)	208 832 (1.0)	0	0	22 468 (2.9)	22 468 (2.9)
Total	19 280 598 (89.6)	1 953 775 (9.1)	276 789 (1.3)	21 511 161 (100)	579 938 (73.7)	178 059 (22.6)	28 721 (3.7)	786 718 (100)

Abbreviations: CKD, chronic kidney disease; eGFR, estimated glomerular filtration rate; NHANES, National Health and Nutrition Examination Surveys; VA, Veterans Affairs.

^a^ Estimated for the US Black population. Data are weighted.

^b^ Individuals who would be classified as having a more advanced stage of CKD without inclusion of the race coefficient.

## Discussion

In this cross-sectional study, removal of the coefficient for Black race from the CKD-EPI equation was associated with a substantial increase in the estimated prevalence of CKD among the US Black population and among US Black veterans who use a large nationally integrated health system. The main limitation of this study is the inconsistency in the reporting of race across all study participants. In addition, the potential implications of our findings for the outcomes of Black individuals in the US (eg, use of health care services) were beyond the scope of this research letter. A rigorous examination of the consequences of this large, expected shift in the estimated burden of CKD is required, with sensitivity to individual patient perspectives and public health considerations to minimize the possibility of unintended harm.^6^ Our findings suggest that continuing research to improve current GFR estimating equations using race-neutral biomarkers should be given high priority.
